# Computational Guided Drug Targets Identification against Extended-Spectrum Beta-Lactamase-Producing Multi-Drug Resistant Uropathogenic *Escherichia coli*

**DOI:** 10.3390/biomedicines11072028

**Published:** 2023-07-19

**Authors:** Harpreet Kaur, Vinay Modgil, Naveen Chaudhary, Balvinder Mohan, Neelam Taneja

**Affiliations:** Department of Medical Microbiology, Post Graduate Institute of Medical Education and Research, Chandigarh 160012, India

**Keywords:** extended-spectrum beta-lactamase, urinary tract infections, *Escherichia coli*, multi-drug resistant, subtractive genomics, drug targets

## Abstract

Urinary tract infections (UTIs) are one of the most frequent bacterial infections in the world, both in the hospital and community settings. Uropathogenic *Escherichia coli* (UPEC) are the predominant etiological agents causing UTIs. Extended-spectrum beta-lactamase (ESBL) production is a prominent mechanism of resistance that hinders the antimicrobial treatment of UTIs caused by UPEC and poses a substantial danger to the arsenal of antibiotics now in use. As bacteria have several methods to counteract the effects of antibiotics, identifying new potential drug targets may help in the design of new antimicrobial agents, and in the control of the rising trend of antimicrobial resistance (AMR). The public availability of the entire genome sequences of humans and many disease-causing organisms has accelerated the hunt for viable therapeutic targets. Using a unique, hierarchical, in silico technique using computational tools, we discovered and described potential therapeutic drug targets against the ESBL-producing UPEC strain NA114. Three different sets of proteins (chokepoint, virulence, and resistance genes) were explored in phase 1. In phase 2, proteins shortlisted from phase 1 were analyzed for their essentiality, non-homology to the human genome, and gut flora. In phase 3, the further shortlisted putative drug targets were qualitatively characterized, including their subcellular location, broad-spectrum potential, and druggability evaluations. We found seven distinct targets for the pathogen that showed no similarity to the human proteome. Thus, possibilities for cross-reactivity between a target-specific antibacterial and human proteins were minimized. The subcellular locations of two targets, ECNA114_0085 and ECNA114_1060, were predicted as cytoplasmic and periplasmic, respectively. These proteins play an important role in bacterial peptidoglycan biosynthesis and inositol phosphate metabolism, and can be used in the design of drugs against these bacteria. Inhibition of these proteins will be helpful to combat infections caused by MDR UPEC.

## 1. Introduction

The World Health Organization (WHO) has listed AMR as one of the top ten worldwide public health concerns to humanity in the twenty-first century [1]. Recently, AMR has been referred to as the “silent tsunami facing modern medicine” [2]. The heightened use/misuse of antibiotics in human medicine and animal agriculture primarily contributes to this phenomenon. An alarming increase of AMR in bacteria causes community or hospital-acquired infections. Of particular interest is the multi-drug resistant (MDR) pathogen *Escherichia coli (E. coli)*, which is the most common causative pathogen in many types of infections, especially in countries with poor healthcare systems [3]. *E. coli* are part of normal human and animal gut flora, but are also the predominant cause of community and hospital-acquired UTIs. AMR among UPEC has significantly grown recently, restricting the choice of treatment options. Special notes are ESBL-producing *E. coli* (ESBL-EC), which are responsible for severe human morbidity and mortality, with significant economic losses and disease burdens [4]. ESBLs are a fast-growing class of beta-lactamases that can hydrolyze oxy-imino cephalosporins (ceftriaxone, cefuroxime, ceftazidime, cefepime, and cefotaxime) and monobactams which results in drug resistance [5]. Thus, these bacteria have become resistant to many available antibiotics, and may also be resistant to drugs of last resort such as carbapenems [6]. The use of carbapenems has dramatically increased over the previous few years, leading to carbapenem-resistant *Enterobacteriaceae* (CRE), against which hardly any antibiotics are available, except for colistin [6]. ESBLs are rampant in low and middle-income countries (LMICs) [4]. WHO has put ESBL-EC on its priority list against which new therapeutics are being developed [7].

The accessibility of both the human and microbial genomes has made it easier to apply comparative and subtractive genomics approaches, in which the genomes of the human host and pathogens are compared and homologous host proteins are identified as non-targets. Pathogen-specific proteins with various therapeutic properties have been discovered [8,9,10]. Such approaches have been used to identify non-homologous proteins as potential drug targets in many pathogens, including *Staphylococcus saprophyticus, Mycobacterium avium, Acinetobacter baumannii*, *Pseudomonas aeruginosa, Burkholderia pseudomallei,* and *Streptococcus pneumoniae* [9,11,12,13]. These approaches, combined with the advancements in computational biology and the availability of diverse bioinformatics tools, are revolutionizing fields of drug discovery and design by minimising time and expense of wet-lab screening [13,14]. 

In this study, we applied a subtractive genomics approach using an ESBL- producing MDR UPEC strain NA114. This UPEC strain was identified in Pune, India, in the urine of a 70-year-old man who had prostatitis. It is an MDR pathogen and ESBL-producing strain, resistant to amoxicillin, co-trimoxazole, tetracycline, gentamicin, nalidixic acid, ciprofloxacin, ceftazidime, and nitrofurantoin [15]. To identify and select the proteins that are crucial for the pathogen’s survival, we made use of a variety of computational techniques, software, and web databases. Only proteins specific to pathogens were shortlisted, thereby avoiding host homology. In order to identify possible broad-spectrum therapeutic targets, we considered all functional metabolic pathways, virulence genes, and resistance genes and discovered seven distinct targets that are unique to UPEC. Furthermore, as they showed no similarity to the human proteome, the possibilities of cross-reactivity between a new drug that uses one of these targets and human proteins were minimized. The subcellular locations of two targets, ECNA114_0085 and ECNA114_1060, were predicted as cytoplasmic and periplasmic, respectively. These proteins play an important role in peptidoglycan biosynthesis and inositol phosphate metabolism. Therefore, the development of new drugs against these targets could be promising steps towards eliminating UTIs caused by *E. coli.*

## 2. Material and Methods

**Figure 1:**
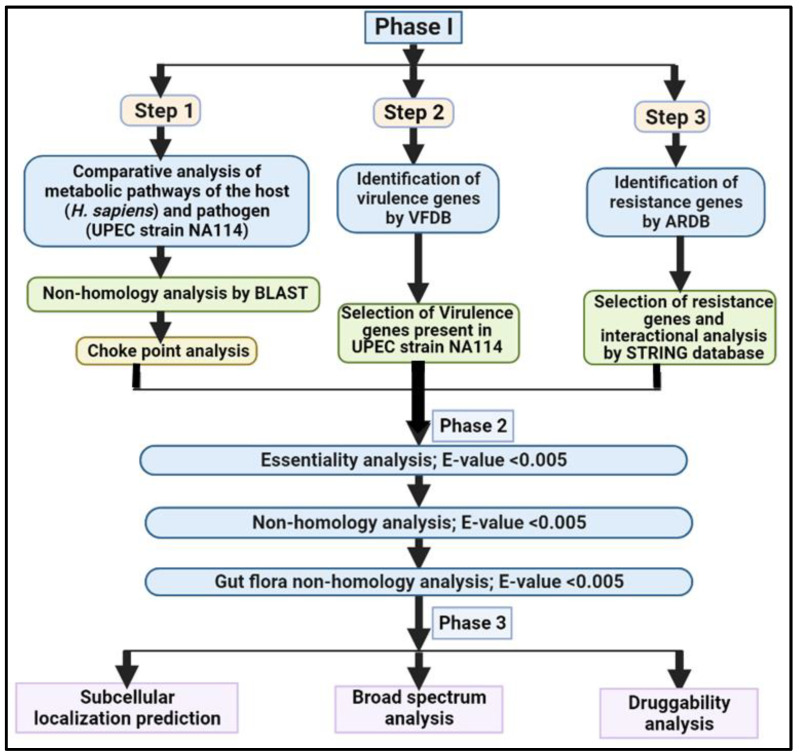
Complete workflow of the study.

Figure 1 displays the full workflow that was employed in this study. 

### 2.1. Phase I: Comparative Analysis of Pathogen and Human Proteome

#### Step1: Non-Homology Analysis

The Kyoto Encyclopedia of Genes and Genomes (KEGG) database was used to compare the pathways of the host *Homo sapiens* (*H. sapiens*) and the UPEC strain NA114 [16]. Pathways that are not present in the human host were considered to be distinct pathways. All proteins in the pathways were searched using the protein basic local alignment search tool (BLASTp) against the non-redundant database. Non-homologous proteins were chosen based on rigorous measurements showing no similarity [17].

### 2.2. Chokepoint Analysis

In a metabolic network, a “chokepoint reaction” is a reaction that consumes or creates a single substrate or product. The inhibition of enzymes involved in chokepoint reaction will hamper the important cell function [18]. Chokepoint analysis is carried out using simple statistics. The confidence level (CL) of all non-homologous proteins (enzymes) has been calculated by using the simple formula [19],

Confidence level of an enzyme=No. of pathways in which an enzyme is found as a choke point/Total no. of pathways of the enzyme×100.

Proteins with confidence scores below 50% were removed, while those with confidence scores above 50% were chosen for further study.

#### 2.2.1. Step 2: Analysis of Virulence Genes 

Virulence factors (VF) have been identified as prospective therapeutic targets in drug development. The pathogen would become avirulent if these virulence proteins were inhibited, as these proteins are essential for the establishment and severity of infection [20]. The virulence factor database (VFDB) contains virulence genes from different bacterial species, including *E. coli* [21]. The VFDB was used to compile a comprehensive list of virulence genes, and corresponding sequences of these genes were taken from the national center for biotechnology information (NCBI)/KEGG database. 

#### 2.2.2. Step 3: Analysis of Resistance Genes

In a survey in India, resistance rates of UPEC to various antibiotics were reported for gentamicin (58.2%), beta-lactams (57.4%), quinolones (74.5%), amikacin (33.4%), nalidixic acid (77.7%), co-trimoxazole (48.5%) and cefuroxime (56%) [22]. The genes that produce resistance to popular antibiotics were found using the antibiotic resistance database (ARDB). High-confidence interactors were predicted using the Search Technique for the Retrieval of Interacting Genes/Proteins (STRING) version 10 for each resistant protein [23]. Further, the proteins with high interactions were manually screened for their presence in the human host. 

### 2.3. Phase II: Subtractive Analysis

#### 2.3.1. Analysis of Essential Genes

We identified potential therapeutic drug targets for powerful essential proteins using a BLASTp search against the database of essential genes (DEG) [24]. DEG, a database of essential genes (genes required for an organism’s survival), contains essential genes from both eukaryotic and prokaryotic organisms. Protein alignments with an e-value of 0.005 or less were considered more meaningful. The resultant proteins were reconstituted and used as input for the non-homology analysis.

#### 2.3.2. Non-homology Analysis

The goal of the non-homology study is to identify proteins specific to the pathogen that are non-homologous to the human host. This process is important because it prevents the drug from binding to any homologous host proteins and reduces the drug’s undesired cross-reactivity [25]. All short-listed proteins were analysed through a BLASTp against a non-redundant *H. sapiens* database [17,25,26]. Proteins not present in the human host were chosen for the following phase.

#### 2.3.3. Human Gut Flora Non-homology Analysis

Shortlisted proteins from the prior step were compared to the proteome of human gut microflora. A normal healthy human’s gastrointestinal tract contains approximately 1014 bacterial species [27]. The gut microbiota has a symbiotic relationship with the host, and it plays an important role in metabolism by digesting food particles and protecting the gut against harmful bacteria invasion [28]. Unintentional inhibition of gut flora proteins can degrade the microbiota, resulting in negative consequences for the host. To avoid pharmacological cross-reactivity with gut microflora proteins, the target proteins were homologized against the gut flora proteome using BLASTp with an expected value (e-value) of 0.005 [26]. The analysis included a list of gut microorganisms reported in the literature [29]. The proteins with no more than ten matches on their own were chosen. The potential target proteins were further analyzed for their quantitative characterization.

### 2.4. Phase III: Quantitative Characterization of Putative Drug Targets 

#### 2.4.1. Subcellular Localization Prediction

*E.coli* is a gram-negative bacterium that possesses an outer cell membrane. Therefore, proteins after synthesis were localized in five possible locations, namely, cytoplasm, periplasm, and the plasma, extracellular, and outer membranes. The localization analysis aims to identify a protein as a potential therapeutic or vaccination target. Surface membrane proteins can be employed as vaccination targets, while cytoplasmic/periplasmic proteins can be exploited as therapeutic drug targets [30]. Once the potential targets were selected, identification of the location of these proteins was attempted to know their functional assignment. For the identification of sub-cellular locations, the Cell Ontology-based Classification (Cello) tool [31], PSORTb (https://www.psort.org/psortb/) [32], and a program for identification of sub-cellular localization of bacterial proteins (ProtCompB) were used. 

#### 2.4.2. Broad-Spectrum Analysis 

For the identification of broad-spectrum targets, potential therapeutic targets were examined using a BLASTp search against a large number of pathogenic microorganisms [29]. A list of pathogenic bacteria involved in UTIs reported in the virulence factor database (VFDB), and pathogenic bacteria from the literature, as reported by Shanmugham et al. 2013 were considered in this analysis [21]. According to the homology analysis for each pathogen, close homologs are more likely to represent a “promising broad-spectrum target” if they are found in a larger number of pathogenic species.

#### 2.4.3. Druggability Analysis 

Further, we performed a BLASTp search against the DrugBank database with an e-value of 0.005 to assess each protein’s drug-ability potential. The Drug Bank database is a one-of-a-kind resource that combines pharmacological data with information on drug targets at the sequencing, structure, and pathway levels [33]. 

## 3. Results 

### 3.1. Phase I: Comparative Analysis

The metabolic pathways of the host *H. sapiens* and the UPEC have been compared *in silico*. Pathways that are not found in humans but are found in pathogens are referred to as distinct pathways. The 119 metabolic pathways considered in this study are linked to nucleotide, carbohydrate, amino acid, or vitamin metabolism, or cofactor biosynthesis. Thirty four of the 119 pathways have been identified as distinct. Non-homologous proteins were identified in 30 of the 34 pathways.

#### 3.1.1. Chokepoint Enzymes

Chokepoint analysis was investigated using non-homologous proteins from several metabolic pathways. Thirty-one of the 62 proteins were identified as choke point enzymes, with a confidence level of more than 50%, and were chosen for further analysis (Table 1). 

#### 3.1.2. Virulence Factors Analysis

Several types of virulence factors, including adherence, iron uptake-aerobactin, and hemolysin are enlisted in VFDB. In UPEC, 69 virulence genes were found. Out of 69, nineteen were found in UPEC strain NA114 (Table 2).

#### 3.1.3. Resistance Gene Analysis 

Resistance genes were taken from the ARDB. In the UPEC strain used in this study, we found 1805 resistance genes. These proteins were further screened from the STRING database, which resulted in 20 genes with high interactions. Out of these 20, six proteins were present in UPEC and not found in humans (Table 3). Protein sequences of these resistance-causing proteins were taken from KEGG/NCBI database. Generally, resistance-causing proteins and their related proteins are thought to be ‘promising therapeutic targets’, because inhibiting them could stop the drug resistance mechanism from working.

### 3.2. Phase II: Subtractive Channel of Analysis

#### 3.2.1. Analysis of Essential Genes

Using the DEG server, and a threshold e-value of 0.005, the combined proteins from phase 1 were further screened for essential genes. The most important condition for a prospective therapeutic target is that it must be a protein that the organism needs to survive. Fifteen of the 52 input proteins identified in phase I were found to be required for the pathogen’s survival and growth (Table 4). Proteins that did not match against any in the DEG database were regarded as non-essential and excluded from the study. The selectivity/specificity of the proteins were analyzed by finding proteins that are non-homologous to the human and gut microbiota proteome.

#### 3.2.2. Non-homology Analysis

The use of proteins that are homologous to the host as therapeutic targets could hurt the host’s metabolism. As a result, several in silico drug-target-discovery algorithms involving filtering out proteins homologous to the human proteome were utilized as the initial step. Non-homology analysis was performed on the short-listed protein datasets. BLASTp was used to look for similarities between the fifteen UPEC proteins and the entire proteome of *H. sapiens* (host). Five proteins were found to be homologous to a human host and excluded from the study. The remaining ten non-homologous proteins were used in further studies.

#### 3.2.3. Gut Flora Non-homology Analysis 

The resulting list of proteins that are non-homologous to *H. sapiens* was screened against the whole proteome microbiota and other gut flora organisms found in the literature using the BLASTp search [29]. From gut flora non-homology analysis, three proteins were found as homologs in microbiota and other gut flora organisms and excluded from the study. The remaining seven proteins were further assessed as putative drug targets.

### 3.3. Phase III

In this phase, the putative drug targets were further analyzed for their properties, namely, cellular location, broad-spectrum, and druggability analysis.

#### 3.3.1. Subcellular Localization Prediction of Putative Targets

The sub-cellular location was predicted by using ProtCompB, Cello, and PSORTb tools. ProtCompB predicts the localization of proteins at the subcellular level in gram-negative bacteria; by using these, five of the seven proteins were found in the inner membrane, one in the periplasm, and one in the cytoplasm. Cello predicts the localization of protein domains using a multi-class support vector machine (SVM) system based on the physicochemical properties of proteins and predicted the location of one protein in the cytoplasm, one in the periplasm, and five in the inner membrane. PSORTb is another tool for the prediction of protein location for gram-positive strains, gram-negative strains, and archaeal sequences. In our dataset, it returned five proteins as cytoplasmic-membrane, and two as cytoplasm. By comparing the results from different tools, the location of five proteins was found in the inner membrane, one in the cytoplasm and one in the periplasm (Table 5). 

#### 3.3.2. Broad-Spectrum Analysis 

Comparative genomic analysis of the screened targets, using a clinically significant *E. coli* strain that causes UTIs described in the VFDB as reference, allowed for an efficient assessment of potential broad-spectrum therapy targets. The broad-spectrum investigation used a list of 240 pathogenic pathogens. A promising broad-spectrum target was discovered using a BLASTp homology search against the entire proteome of each of these bacterial pathogens. The homology search revealed that the screened targets had close homologs in multiple pathogenic species, indicating that *E. coli* proteins are multispecies. Therapeutic compounds that inhibit such broad-spectrum targets could help to eradicate UPEC infections and could be used as possible UPEC-specific drug targets. Such pathogen-specific targets could help to lower the possibility of treatment resistance in a variety of infections. All proteins had more than 70 matches, indicating that the therapeutic targets have a broad spectrum.

#### 3.3.3. Druggability Analysis 

The druggability of the short-listed candidate drug targets was assessed by utilizing a sequence-similarity search against the Drug Bank target database. All seven potential drug targets were searched against the Drug Bank target database manually, which includes US Food and Drug Administration (FDA)-approved drugs, revealing that ECNA114_0085 is homologous to a known target, D-Alanine-D-Alanine-ligase. None of the other targets exhibited any matches to the drug target database (Table 6).

## 4. Discussion

In this study, we used a broad approach and included all functional biosynthetic pathways, such as the metabolism of carbohydrates, energy, amino acids, vitamins, terpenoids, and ketides, as well as those for the biodegradation of xenobiotics. We identified distinct non-homologous pathways that are only found in pathogens and not in human hosts, and the proteins found in these pathways were classified as distinct proteins [34]. Out of 34 considered pathways, non-homologous proteins were found in 30 pathways. However, the fact that they are distinct from, or unrelated to human hosts does not qualify them as effective therapeutic drug targets, as isoenzymes or paralogs exist [35]. To remove this bias, a chokepoint analysis was done. A chokepoint reaction consumes or creates a particular product [18]. Therefore, inhibiting an enzyme that uses a harmful substrate may result in the accumulation of toxic products. Alternatively, if the inhibited enzyme is involved in the creation of a special product, inhibiting it would starve the cell, which may ultimately impair vital cell processes [36]. In light of this, enzymes implicated in chokepoint reactions may be crucial for infectivity and thus, may represent a potential therapeutic target for drug discovery. Thirty-one enzymes from the 62 non-homologous enzymes identified in various metabolic pathways were recognized as chokepoint enzymes. In this work, we have also taken UPEC virulence factors into consideration. Targeting virulence factors has two benefits: first, it will reduce pathogenicity; and second, selection pressure will be minimized, preventing the development of drug resistance. Cinnamaldehyde, baicalein, naringenin, and catechin are a few of the recently identified anti-virulence substances that act by preventing the initiation of quorum sensing (QS) and biofilm production [37,38]. In our study, UPEC strain NA114 was found to contain 19 virulence factors, including those involved in lysine degradation, ABC transporters, biosynthesis of siderophores, two-component pathways, and bacterial secretion systems. By stymieing the actions of proteins linked to antibiotic resistance proteins, drug resistance can also be avoided. To identify potential therapeutic targets for UPEC NA114, we thus included antibiotic resistance genes as targets in the current investigation. Similar combinatorial approaches have been used for the identification of putative drug targets for *Mycobacterium abscessus* where 40 targets were identified [29]. Out of 15 proteins found in the DEG database deemed to be essential for the survival of UPEC, further non-homology and gut flora non-homology analysis yielded seven putative drug targets (Table 3). Since the eligibility and effectiveness of a protein as a therapeutic target depends on where it is located in the cell, in silico tools were employed to predict the subcellular location of the protein [13,39]. Proteins found in the membrane are not ideal as therapeutic targets due to their difficulty in purification and testing. We found two potent druggable proteins (ECNA114_0085 and ECNA114_1060) as promising targets in UPEC strain NA114.

The cytoplasmic ECNA114_0085 protein, identified as a potential target here, is a D-alanine-D-alanine ligase (Ddl) involved in the synthesis of peptidoglycan [9,40]. The peptidoglycan precursor UDPMurNAc-pentapetide contains the terminal dipeptide D-Ala-D-Ala, which is an essential building component for peptidoglycan cross-linking, which provides stability to the cell wall. Therefore, blocking the enzyme Ddl can dramatically reduce the strength of the cell wall, which causes the bacteria to be osmotically lysed. According to a prior study, Ddl is an attractive target for the discovery of drugs in *Mycobacterium tuberculosis* [41]. Vancomycin, which acts only on gram-positive bacteria, binds to Ddl, which inhibits glucosyltransferase, thereby preventing the polymerization and synthesis of N-acetylglucosamine (NAG) and N-acetylmuramic acid (NAM) throughout the peptidoglycan layer [42]. The essential penicillin-binding proteins (PBPs), which are enzymes involved in the final stages of peptidoglycan cross-linking in both gram-negative as well as gram-positive bacteria, covalently bind to beta-lactam antibiotics, making them bactericidal substances that prevent the formation of bacterial cell walls. Early intracellular phases of cell wall synthesis have received little attention as prospective therapeutic targets so far, in contrast to the extracellular stages of peptidoglycan biosynthesis, which are inhibited by beta-lactam and glycopeptide antibiotics [43]. Currently, only two drugs are in use to block intracellular peptidoglycan synthesis: D-cycloserine, which blocks both Ddl and alanine racemase, and fosfomycin, a MurA ligase inhibitor. The internal peptidoglycan precursor UDPMurNAc-pentapeptide is assembled by the mur ligases MurC, MurD, MurE, and MurF by the sequential addition of L-Ala, D-Glu, m-Dpm or L-Lys, and D-Ala-D-Ala to UDP-MurNAc. Ddl is responsible for supplying the MurF substrate, D-alanyl-D-alanine [43]. Because cross-linking of peptidoglycan chains takes place between the penultimate D-Ala in a second pentapeptide strand and the C6-NH2-group of meso-diaminopimelic acid in gram-negative bacteria and the NH2-group of pentaglycine in gram-positive *Staphylococcus*, this terminal dipeptide is crucial in the construction of the bacterial cell wall. In both situations, the intrastrand D-Ala-D-Ala bond is broken and a new interstrand peptide bond is formed [43]. This enzyme is common in prokaryotes, but absent in eukaryotes, making it a likely candidate target for antibiotic development. It is possible to design inhibitors against this enzyme, crucial to the peptidoglycan synthesis process, that could cause a loss in the structural integrity of bacterial cell walls and osmotic lysis of pathogenic bacteria.

The periplasmic protein appA (ECNA114_1060) involved in the metabolism of inositol phosphate was found to be another attractive target. Gram-negative bacteria are extremely resistant to the penetration of antimicrobials due to their double-layered cell envelope and a variety of efflux pumps. Proteins in the periplasm are easier to target, since only penetration of the outer membrane is required, whereas cytoplasmic targets are difficult to reach [44]. In addition to assisting in the production of virulence factors, adhesion molecules, and signaling molecules, periplasmic proteins are crucial for sustaining cell viability, cell division, and membrane integrity. All eukaryote genomes contain the inositol polyphosphate production enzymes, yet standard search techniques frequently fail to uncover the amino acid sequence homology of these enzymes [45]. Similarity searching between microbial and human inositol phosphate kinases is restricted to fewer catalytically important residues, which include proline, aspartic acid, lysine, glycine, serine, leucine, tyrosine, isoleucine, histidine, threonine, glutamic acid, and phenylalanine [45]. Recent studies of the Inositol phosphate metabolic pathways in pathogenic protozoa (*Trypanosome brucei*) and fungi (*Cryptococcus neoformans*) are being exploited as promising antimicrobial targets [45]. Inositol phosphate metabolism has been identified as a putative drug target for many bacterial pathogens including *Staphylococcus saprophyticus, Klebsiella pneumoniae*, and *Streptococcus pneumoniae* [11,46,47]. Despite the benefits and optimistic future of comparative genomics in the identification of possible therapeutic targets, there are still certain limitations. Despite the fact that comparative genomics is frequently utilized in the creation of medications to combat drug-resistant bacteria, the failure rate of existing antibiotics is substantially higher than the rate at which new antibiotics are developed. Additionally, antibiotics are transient treatments for infections. The use of comparative genomics in the creation of antibiotics is a long-debated topic due to the fact that their value is significantly lower than medications used in the treatment of chronic diseases [48]. A further problem is that, despite the fact that comparative genomics can decrease the number of experimental targets and identify some appealing proteins as prospective therapeutic targets, the range of potential targets that can be screened by this method is still quite broad and the cost and time used in the development process is prohibitive. The majority of these putative targets identified by comparative genomics are unlikely to be validated through experiments. Therefore, it would be advantageous to integrate network-based approaches with comparative genomics to further limit the range of experimental targets. This will use less time and material, and cut costs during the early stages of drug research and development.

## 5. Conclusions

Using a unique hierarchical in silico technique, we discovered and described two potent druggable proteins as prospective therapeutic drug targets against ESBL-producing MDR UPEC strain NA114. The identified drug targets are distinct and have the potential to be exploited for designing new antimicrobial agents against UPEC. The computer-aided drug design method can also be utilized to identify homologous compounds for these targets. The search for possible therapeutic targets in ESBL-producing MDR UPEC strain NA114 was directed by comparative and subtractive genomics. Essentiality, non- homology to the human host, availability in the drug bank, and sub- cellular location were used to narrow down the list of targets in this study. Our research uncovered prospective therapeutic targets (ECNA114_0085 and ECNA114_1060) that are critical in the treatment of *E. coli* urinary tract infections. 

## Figures and Tables

**Table 1:** List of chokepoint enzymes from different pathways.

Enzyme ID	Total No. of Pathways of the Enzyme
ECNA114_0085	ena00473, ena00550, ena01100, ena01502
ECNA114_3261	ena00550
ECNA114_1004, ECNA114_3778	ena00540, ena01100
ECNA114_2045	ena01053
ECNA114_0627	ena00010, ena00030, ena00052, ena00230, ena00500, ena00520, ena00521, ena01100, ena01110, ena01120, ena01130
ECNA114_2137, ECNA114_2136	ena00521, ena00523, ena01100, ena01130
ECNA114_2904, ECNA114_2317	ena00071, ena00280, ena00310, ena00362,00380, ena00620, ena00630, ena00640,ena00650, ena00900, ena01100, ena01110, ena01120, ena01130, ena01200, ena01220, ena01212
ECNA114_1060	ena00562, ena00627, ena01120
ECNA114_3742	ena00910, ena01120, ena02020
ECNA114_1411	ena00010, ena00071, ena00350, ena00625, ena00626, ena00650, ena01100, ena01110, ena01120, ena01130, ena01220
ECNA114_1698, ECNA114_1052, ECNA114_3080	ena00633, ena01120
ECNA114_3742, ECNA114_3652	ena00010, ena0071, ena00350,ena00625, ena00626, ena01100, ena01110, ena01120, ena01130, ena01150
ECNA114_4463	ena02060, ena00500
ECNA114_2504	ena00520, ena02060
ECNA114_1862	ena00051, ena00520, ena01100, ena02060
ECNA114_4175, ECNA114_4173, ECNA114_4172, ECNA114_2735, ECNA114_3748, ECNA114_2977	ena00051, ena02060
ECNA114_3218, ECNA114_3216, ECNA114_3217, ECNA114_3224,	ena00052, ena02060

**Table 2:** List of virulence genes.

Virulence Factors UPEC	Genes Name	Found in UPEC Strain NA114 and Not in Human
Iron uptake	iutA, iucA, iucB, iucC, iucD	iucA, iucB, iucD, iucC
Chu (*E. coli* hemin uptake)	chuA, chuS, chuT, chuU, chuV, chuW, chuX, and chuY	chuU, chuW
Enterobactin	entA, entB, entC, entD, entE, entF, fepA, fepB, fepC, fepD, fepE, and fepG	fepA, fepB, fepC, fepD, fepG, entE, entA, entB, entF, entC
IroN	iroN	iroN
Hemolysin	hlyA, hlyB, hlyC, and hlyD	hlyB, hlyD

**Table 3:** List of selected resistance genes.

Resistance Gene Found in String after ARDB Tool	Found UPEC Strain NA114	Found in Human	Enzyme No.
acrB	Yes	No	c0580
acrA	Yes	No	c0581
macB	Yes	No	c1016
arnA	Yes	No	c2797
tolC	Yes	No	c3781
bacA	Yes	No	c3807

**Table 4:** List of selected essential genes.

Sr. No.	Query Protein	No. of a Homolog in DEG	DEG Accession Number
1	ECNA114_0085	1	DEG10180021
2	ECNA114_1004	2	DEG10190079, DEG10180150
3	ECNA114_3778	3	DEG10480267,DEG10180536, DEG10190246
4	ECNA114_2045	1	DEG10180357
5	ECNA114_2137	1	DEG10180210
6	ECNA114_3652	1	DEG10180338
7	ECNA114_1052	1	DEG10180159
8	ECNA114_1862	1	DEG10180464
9	ECNA114_3218	1	DEG10180032
10	entD	1	DEG10190060
11	entE	1	DEG10180357
12	fepB	1	DEG10180106
13	fepC	2	DEG10190239; DEG10480100
14	arnAc2797	3	DEG10180489, DEG10480227, DEG10190203
15	ECNA114_0580	1	DEG10480311

**Table 5:** Predicted subcellular locations of putative drug targets.

Protein Enzyme Code	Cello	PSORTb	ProtCompb	Subcellular Location
ECNA114_4463	Innermembrane	Cytoplasmic Membrane	Innermembrane	Innermembrane
ECNA114_4172	Innermembrane	Cytoplasmic membrane	Innermembrane	Innermembrane
ECNA114_2735	Innermembrane	Cytoplasmic membrane	Innermembrane	Innermembrane
ECNA114_3216	Innermembrane	Cytoplasmic membrane	Innermembrane	Innermembrane
ECNA114_3224	Innermembrane	Cytoplasmic membrane	Innermembrane	Innermembrane
ECNA114_0085	Cytoplasmic	Cytoplasmic	Cytoplasmic	Cytoplasmic
ECNA114_1060	Periplasmic	Cytoplasmic	Periplasmic	Periplasmic

**Table 6:** List of identified putative drug targets.

Putative Targets ID’s	Function	Pathways Involved	Druggability Analysis	Subcellular Location
ECNA114_4463	treB, PTS system,	Phosphotransferase system, Starch and sucrose metabolism	No	Innermembrane
ECNA114_4172	PTS system	Mannose and fructose metabolism, Metabolic pathways, Phosphotransferase system	No	Innermembrane
ECNA114_2735	srlE, PTS system	Mannose and fructose metabolism, Metabolic pathways, Phosphotransferase system	No	Innermembrane
ECNA114_3216	agaW, component of PTS system	Galactose metabolism, Metabolic pathways, Phosphotransferase system	No	Innermembrane
ECNA114_3224	agaD, a component of the PTS system	Galactose metabolism, Metabolic pathways, Phosphotransferase system	No	Innermembrane
ECNA114_0085	D-alanine-D-alanine ligase	D-alanine metabolism, Metabolic pathways, Vancomycin resistance, Peptidoglycan biosynthesis	Yes	Cytoplasmic
ECNA114_1060	appA, Phosphoanhydride phosphohydrolase	Inositol phosphate and riboflavin metabolism, Metabolic pathways	No	Periplasmic

## Data Availability

Not applicable.
